# Foscan® (mTHPC) photosensitized macrophage activation: enhancement of phagocytosis, nitric oxide release and tumour necrosis factor-α-mediated cytolytic activity

**DOI:** 10.1038/sj.bjc.6690648

**Published:** 1999-09

**Authors:** S Coutier, L Bezdetnaya, S Marchal, V Melnikova, I Belitchenko, J L Merlin, F Guillemin

**Affiliations:** Unité de Recherche en Thérapie Photodynamique, Centre Alexis Vautrin, Brabois 54511, Vandœuvre-les-Nancy Cedex, France

**Keywords:** photodynamic, mTHPC, macrophages, phagocytosis, TNF-α, nitric oxide

## Abstract

Photodynamic activation of macrophage-like cells contributes to an effective outcome of photodynamic therapy (PDT) treatment. The possibility for an enhancement of macrophage activity by photosensitization with meta-tetra(hydroxyphenyl)chlorin (mTHPC) (1 μg ml^−1^) was studied in U937, monocyte cell line differentiated into macrophages (U937Φ cells). Phagocytic activity of U937Φ cells was evaluated by flow-cytometry monitoring of ingestion of fluorescein-labelled *Escherichia coli* particles. Increase in irradiation fluence up to 3.45 mJ cm^−2^ (corresponding irradiation time 15 s) resulted in significant increase in fluorescence signal (145%), while at higher light fluences the signal lowered down to the untreated control values. A light energy-dependent production of tumour necrosis factor-alpha (TNF-α) by photosensitized macrophages was further demonstrated using the L929 assay. The maximum TNF-α mediated cytolysis was observed at 28 mJ cm^−2^ and was 1.7-fold greater than that in control. In addition, we demonstrated a fluence-dependent increase in nitric oxide (NO) production by mTHPC-photosensitized macrophages. NO release increased gradually and reached a plateau after irradiation fluence of 6.9 mJ cm^−2^. Cytotoxicity measurements indicated that the observed manifestations of mTHPC-photosensitized macrophage activation took place under the sublethal light doses. The relevance of the present findings to clinical mTHPC-PDT is discussed. © 1999 Cancer Research Campaign


British Journal of Cancer (1999) 81(1), 37–42
© 1999 Cancer Research Campaign
Article no. bjoc.1999.0648

### Foscan® (mTHPC) photosensitized macrophage activation: enhancement of phagocytosis, nitric oxide release and tumour necrosis factor-α-mediated cytolytic activity

**S Coutier, L Bezdetnaya, S Marchal, V Melnikova, I Belitchenko, JL Merlin and F Guillemin**

Unité de Recherche en Thérapie Photodynamique, Centre Alexis Vautrin, Avenue de Bourgogne, Brabois, 54511 Vandœuvre-les-Nancy Cedex, France

**Summary** Photodynamic activation of macrophage-like cells contributes to an effective outcome of photodynamic therapy (PDT) treatment. The possibility for an enhancement of macrophage activity by photosensitization with meta-tetra(hydroxyphenyl)chlorin (mTHPC) (1 μg $ml^{-1}$) was studied in U937, monocyte cell line differentiated into macrophages (U937Φ cells). Phagocytic activity of U937Φ cells was evaluated by flow-cytometry monitoring of ingestion of fluorescein-labelled *Escherichia coli* particles. Increase in irradiation fluence up to 3.45 mJ $cm^{-2}$ (corresponding irradiation time 15 s) resulted in significant increase in fluorescence signal (145%), while at higher light fluences the signal lowered down to the untreated control values. A light energy-dependent production of tumour necrosis factor-alpha (TNF-α) by photosensitized macrophages was further demonstrated using the L929 assay. The maximum TNF-α mediated cytolysis was observed at 28 mJ $cm^{-2}$ and was 1.7-fold greater than that in control. In addition, we demonstrated a fluence-dependent increase in nitric oxide (NO) production by mTHPC-photosensitized macrophages. NO release increased gradually and reached a plateau after irradiation fluence of 6.9 mJ $cm^{-2}$. Cytotoxicity measurements indicated that the observed manifestations of mTHPC-photosensitized macrophage activation took place under the sublethal light doses. The relevance of the present findings to clinical mTHPC-PDT is discussed.

**Keywords:** photodynamic; mTHPC; macrophages; phagocytosis; TNF-α; nitric oxide

Photodynamic therapy (PDT) is a therapeutic modality used for the treatment of a variety of solid neoplasms. The concept of PDT is based on the selective uptake of a photosensitizing chemical in tumour tissue followed by an irradiation of tumours with visible light (Henderson and Dougherty, 1992). The treatment results in a cascade of oxidative events causing cell death and eventual tumour ablation (Pass, 1993).

There are numerous indications in the literature on the contribution of immune reactions to cancer cell inactivation during PDT (Korbelik and Krosl, 1996; Dougherty et al, 1998). These reactions rely on the activation in the early phase (during and immediately after light treatment) of non-specific immune effector cells, such as monocytes/macrophages, neutrophils and mast cells (Krosl et al, 1995). Tumouricidal activity of these activated inflammatory cells, among which the macrophages receive a particular attention, leads to the development of the tumour-specific immunity on the later stages (Yamamoto et al, 1992*b*). Increased macrophage activity after PDT in vitro and in vivo is well documented. The enhancement of Fc-receptor-mediated phagocytosis of macrophages exposed to the pg concentrations of haematoporphyrin derivative or Photofrin and low light fluences was reported by Yamamoto et al (1991). Same authors demonstrated the enhanced ingestion activity of macrophages subjected to small doses of cyanine dyes lumin and platonin and white fluorescent light (Yamamoto et al, 1992*a*; Nakagawa et al, 1993). Increased activity of peritoneal (Yamamoto et al, 1991) and tumour-associated macrophages (Krosl et al, 1995) was also reported after in vivo PDT.

Macrophages upon activation have been shown to produce different cytotoxic species including proteases, reactive oxygen intermediates, tumour necrosis factor alpha (TNF-α) and reactive nitrogen species such as nitric oxide (NO) (Adams and Nathan, 1983; Albina and Reichner, 1998). PDT also results in a release of different cytokines and oxidants from the macrophages and cells in culture. For instance, peritoneal murine macrophages, when sensitized by Photofrin II and exposed to light, may be stimulated to produce TNF-α (Evans et al, 1990). The up-regulation of TNF-α production in keratinocytes in vitro by PDT using a phthalocyanine-derived photosensitizer has been reported (Anderson et al, 1993). Recently, NO production was demonstrated in phthalocyanine-photosensitized tumour cells (Gupta et al, 1998). Both TNF-α and NO have numerous physiological functions and, besides direct tumouricidal action, they might affect tumour microvasculature resulting in tumour regression (Asher et al, 1987; Korbelik et al, 1998).

Meta-tetra(hydroxyphenyl)chlorin (mTHPC, Foscan®) is a second generation photosensitizer that is currently being evaluated for anti-neoplastic activity. Phase II/III clinical trials are in progress for the treatment of patients with diffuse malignant mesothelioma (Ris et al, 1991), primary head and neck squamous cell carcinoma (Dilkes et al, 1997) and upper aerodigestive tract cancers (Grosjean et al, 1993). The possibility for macrophage activation upon photosensitization with mTHPC has not yet been studied.

*Received 14 December 1998*
*Revised 11 March 1999*
*Accepted 18 March 1999*

*Correspondence to:* L Bezdetnaya

The above considerations prompted us to investigate in this work the effect of mTHPC photosensitization on the phagocytic capacity, TNF-α production and NO release by the macrophages. The experimental model used was U937 human monocyte cell line, induced to differentiate into macrophages.

### MATERIALS AND METHODS

### Photosensitizer mTHPC (Foscan®) was kindly provided by Scotia Pharmaceuticals Ltd (Guildford, UK). Stock solution was made according to manufacturer's recommendation (30% polyethylene glycol 400, 20% ethanol and 50% water). Further dilution was performed in phosphate-buffered saline (PBS; Dutscher, Brumath, France) with the addition of 2% heat-inactivated fetal calf serum (FCS; Dutscher, France). Final mTHPC concentration was 1 μg $ml^{-1}$.

### Cell cultures

Mouse fibroblast L929 cells and human histocytic lymphoma U937 cells (European Collection of Cell Cultures, UK) were cultured in RPMI-1640 medium (Gibco, France), supplemented with 10% heat-inactivated FCS, 1% streptomycin–penicillin (Gibco, France) and 200 mM L-glutamin (Gibco, France).

L929 cells growing in confluent cultures were trypsinized to obtain cell suspension. Two hundred microlitres of cell suspension containing $2 \times 10^4$ viable cells were plated per well into 96-well microtitre dishes. Cells were allowed to attach to the dishes for 12 h at 37°C before being used in the TNF-α assay.

The differentiation of U937 cells into the macrophages (U937Φ cells) was induced by 12-*O*-tetradecanoylphorbol-13-acetate (TPA; Sigma), using the modification of an earlier described procedure (Hoff, 1992). U937 cells were placed into the individual wells of the 24-well plates at a seeding density of $2 \times 10^5$ cells $ml^{-1}$. TPA was added into each well from an ethanol stock solution to reach the final concentration of 100 ng $ml^{-1}$ (final ethanol concentration was 1%). Cells were incubated for 48 h at 37°C before being used in the experiments. Majority of the cells attached to the plate's bottom in 24 h.

For the cytotoxicity assay, suspension of U937 cells in growth medium was transferred into 50 ml polypropylene tube. TPA was added to reach a final concentration of 100 ng $ml^{-1}$. After that, 200 μl of cell suspension ($2 \times 10^5$ cells $ml^{-1}$) were plated per well into 96-well microtitre dishes. Incubation conditions were the same as described above.

### PDT of U937Φ cells

Following 48 h incubation, cells were washed three times with PBS and exposed to mTHPC (1 μg $ml^{-1}$). After 3 h incubation at 37°C, medium was removed, cells were washed three times with cold PBS and fresh medium was added before cell irradiation. Irradiation was carried out at 650 nm using a dye laser (Spectra-Physics 375 B), pumped with an argon laser (Spectra-Physics 2020, Les Ulis, France). The output power was 30 mW. Light spot was 13 cm in diameter, providing the fluence rate of 0.23 mW $cm^{-2}$.

### Phagocytosis assay

Rapid microfluorometric phagocytosis assay by using fluorescein-conjugated *E. coli* particles was proposed earlier by Wan et al (1993). We have modified the procedure with the aim of optimization for flow cytometry measurements. Briefly, after light exposure U937Φ cells were incubated with 30 μl of fluorescein isothiocyanate (FITC), fluorescent *E. coli* particle suspension (Vybrant ™ Phagocytosis assay kit, Molecular Probes, Inc., USA), for 1 h at 37°C. *E. coli* suspension was removed from the plate wells and the extracellular fluorescence was quenched by 1 min contact with 100 μl of trypan blue solution (Vybrant™ Phagocytosis assay kit, Molecular Probes, Inc., USA). The trypan blue solution was aspirated and replaced by 100 μl of PBS. Cells were removed from the dish by means of scraping. The intracellular *E. coli* fluorescence was measured by flow cytometry technique. The flow cytometer (Orthocyte, OrthoDiagnostic Systems, Roissy, France) was equipped with a xenon lamp and a filter block for excitation at 488 nm. Fluorescence of FITC was collected through a 530 ± 15 nm-band pass filter. Results were expressed as the relative intracellular fluorescence, which was defined as the ratio of FITC fluorescence intensity in experimental samples to FITC fluorescence intensity in control cultures.

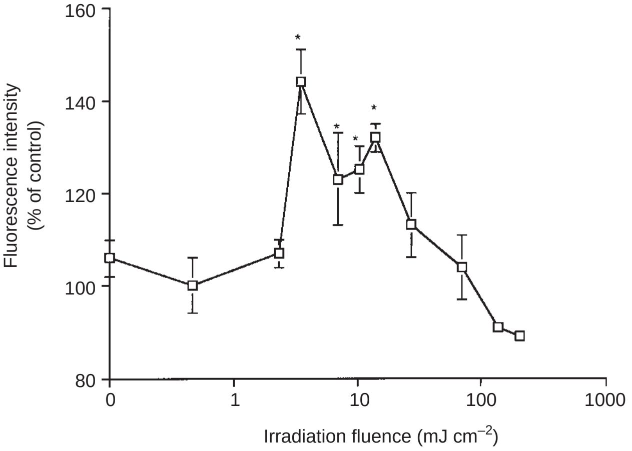

**Figure 1** Phagocytic activity of U937Φ cells exposed to mTHPC (1 μg $ml^{-1}$) and red light (650 nm, 0.23 mW $cm^{-2}$) as a function of irradiation fluence. Phagocytic activity is expressed as a fluorescence intensity of ingested FITC-labelled *E. coli* particles as assessed by flow cytometry (λ em. = 530 nm) immediately after PDT. Each value is the mean of five experiments ± s.e.m. (bars); * $P < 0.05$ when compared with untreated control

### Photocytotoxicity assay

U937Φ cells plated in 96-well dishes were treated photodynamically and were incubated for 18 h at 37°C. Cell survival was assessed by 3-(4,5-dimethylthiazol-2-yl)-2.5-diphenyl-2H-tetrazolium bromide (MTT; Sigma) colourimetric assay, which provides data equivalent to clonogenic assay (McHale et al, 1988; Merlin et al, 1992). Briefly, 50 μl of 0.5% MTT solution were added into each well and incubated for 3 h at 37°C to allow MTT metabolization. After incubation, formazan crystals were dissolved by adding 50 μl of 25% sodium dodecyl sulphate (SDS) solution per well and vigorously pipetted to ensure homogeneity of solution prior to scanning. The absorbance at 540 nm was measured on a Multiskan MCC 340 plate reader (Flow). Appropriate blanks were subtracted from experimental results. The results were given as the percentage of the values obtained with the control cultures.

© 1999 Cancer Research Campaign

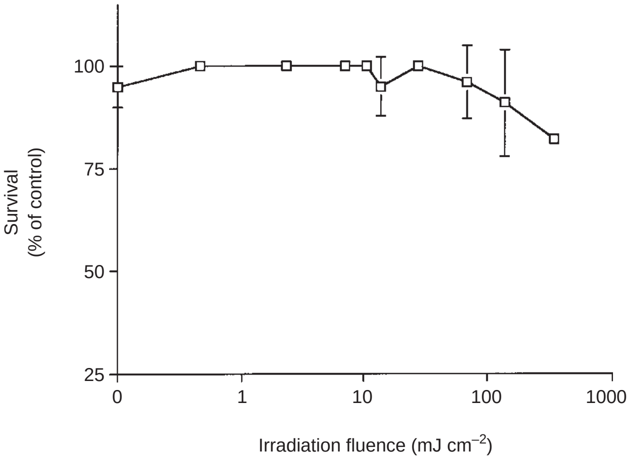

**Figure 2** Photocytotoxicity of U937Φ cells exposed to mTHPC (1 μg ml$^{-1}$) and red light (650 nm, 0.23 mW cm$^{-2}$) as a function of irradiation fluence. Cytotoxicity was determined using MTT colorimetric assay 18 h following PDT. Each value is a mean of three experiments ± s.e.m. (bars)

### Nitrite determination

To assess the production of NO by U937Φ cells, the concentration of nitrite in the culture medium from both the untreated or photodynamically treated cells was measured using the Griess reaction, as described earlier (Sveinbjornsson et al, 1996). Eighteen hours after irradiation, 100 μl of supernatant from U937 cells were incubated at 23°C with 50 μl of Griess reagent (1% sulphanilamide, 0.1% *N*-(1-naphthyl)ethylenediamine dihydrochloride in 2.5% $H_3PO_4$; all obtained from Sigma). After 10 min, absorbance at 550 nm was measured using a Multiskan plate reader. The same experimental conditions (seeding density of $2 \times 10^5$ cells ml$^{-1}$, time intervals) have been applied to untreated control cells (neither photosensitizer nor light). Nitrite concentration was determined by comparing the absorbance with that of standard solutions of sodium nitrite ($NaNO_2$) in RPMI. The results were expressed in percentages of nitrite production in experimental samples compared to that of the untreated control cultures.

### TNF-α-mediated cytolytic activity

Measurement of the bioactivity of TNF-α was based on monitoring the cytolytic effect of U937Φ cell supernatants on the TNF-α responsive cell line, L929 (Evans et al, 1990). The U937Φ cells were treated photodynamically and incubated for 18 h at 37°C. After the incubation, 50 μl of cell supernatant was added to the L929 cells growing in monolayers in sextuplates. Fifty microlitres of a 2 μg ml$^{-1}$ actinomycin D solution (Merck Sharp and Dohme International, USA) was added to each well. In every experiment (untreated cells, cells exposed to photosensitizer alone and photodynamically treated cells) the solution of 1 μg ml$^{-1}$ purified mouse anti-human TNF-α monoclonal antibodies (Pharmingen, USA) was then added to three of the sextuplates to neutralize TNF-α activity. In addition, six wells containing L929 cells received 100 μl of complete medium containing actinomycin D only and served as a control for cell viability in the absence of macrophage supernatant. The plates were incubated for 18 h at 37°C. Cells were then fixed in methanol:acetic acid (3:1) and stained by means of 15-min incubation with 10% ethanol containing 0.5% crystal violet. Plates were washed three times with water and were allowed to dry. The remaining dye was solubilized with 100 μl of a 10% acetic acid and 20% ethanol solution. Plates were shaked slowly and the absorbance of each well was measured at 540 nm with Multiskan microplate reader.

Cytotoxicity was expressed as the absorbance values in experimental wells relative to those in controls measured in the absence of macrophage supernatant. To establish the specificity of the TNF-α-induced cytolysis, the values of cytotoxicity detected in the presence of TNF-α monoclonal antibodies were subtracted from those obtained in their absence. Final results were expressed as relative cytotoxicity, which was defined as the ratio of TNF-α-mediated cytotoxicity in photodynamically treated samples to TNF-α-mediated cytotoxicity in samples exposed to culture medium from untreated macrophages (neither photosensitizer nor light).

### Statistical analysis

The results were expressed as mean ± standard error of the mean (s.e.m.). For the phagocytosis, NO production and TNF-α assays, the comparisons was also made using Mann–Whitney *U*-test. Difference was considered significant when *P*-value was < 0.05.

### RESULTS

### Phagocytic activity of U937Φ cells after mTHPC photosensitization mTHPC loaded U937Φ cells were exposed to increasing doses of light (λ = 650 nm) and brought into contact for 1 h with fluorescein-labelled *E. coli* particles. Macrophage ingestion (phagocytic) activity was then assayed with a flow cytometry technique by taking advantage of the fluorescent properties of bioparticles (λem. = 530 nm). Phagocytic activity of cells in the presence of photosensitizer alone did not differ significantly from control values (cells exposed to medium alone) ($P > 0.05$). Exposure of macrophages to light affected their phagocytic activity in a dose-dependent manner. The fluorescence of ingested bioparticles began to increase after irradiation with the light fluence of 2.3 mJ cm$^{-2}$ (corresponding irradiation time 10 s) and reached a maximum (145%) at 3.45 mJ cm$^{-2}$ (Figure 1). At light fluences up to 14 mJ cm$^{-2}$, phagocytic capacity was significantly higher than in untreated control. Further increase in irradiation fluence led to gradual decrease in phagocytosis. Light fluences greater than 28 mJ cm$^{-2}$ resulted in phagocytosis suppression below the level of untreated control.

### Photocytotoxicity of U937Φ cells

To address the question if suppression of ingestion activity of the macrophages was caused by cytotoxicity, a MTT assay was used for photocytotoxicity measurements. Up to irradiation with the fluence of 0.2 J cm$^{-2}$ no significant cytotoxicity was observed as compared to untreated control (Figure 2). Further increase in irradiation fluence resulted in a progressive decrease in cell survival that reached 82% at 0.35 J cm$^{-2}$ (Figure 2). Therefore, the suppression of phagocytic activity at irradiation fluence of 14 mJ cm$^{-2}$ and above cannot be explained by the irreversible cell photodamage, but may be due to the sublethal cell injury.

© 1999 Cancer Research Campaign

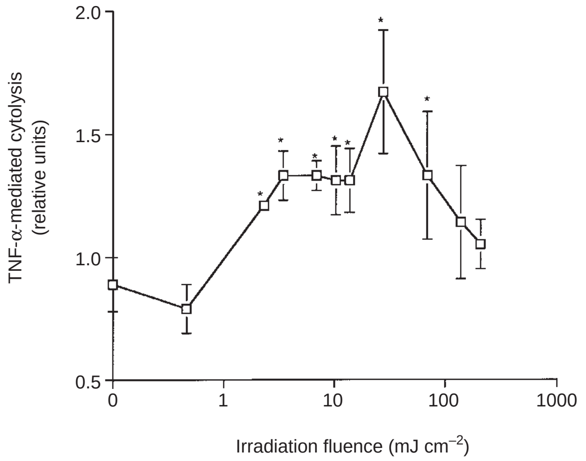

**Figure 3** Biological activity of TNF-α produced by mTHPC-photosensitized U937Φ cells (1 μg $ml^{-1}$; 650 nm, 0.23 mW $cm^{-2}$) as a function of irradiation fluence. The bioactivity of TNF-α was determined based on the cytolytic effects of macrophage supernatants on TNF-α-sensitive murine L929 cells. The number of viable cells was determined by crystal violet colourimetric assay 18 h after PDT. Cytolysis in the presence of the supernatants from untreated macrophages was taken as unity. Each value is the mean of three experiments ± s.e.m. (bars); * $P < 0.05$ when compared with untreated control

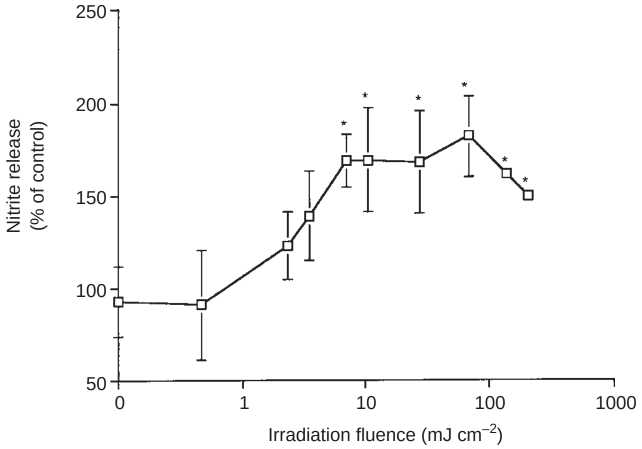

**Figure 4** Nitrite production by U937Φ cells exposed to mTHPC (1 μg $ml^{-1}$) and red light (650 nm, 0.23 mW $cm^{-2}$) as a function of irradiation fluence. The percentage of nitrite in the medium was measured 18 h after PDT. The nitrite level was expressed as percentage of the amount of nitrite produced in untreated cells. Each value is the mean of five experiments ± s.e.m. (bars); * $P < 0.05$ when compared with the control

### Induction of TNF-α-mediated cytolysis by photodynamically treated U937Φ cells

In this section we investigated the possibility for TNF-α production by mTHPC-photosensitized U937Φ cells. Bioactivity of TNF-α was measured monitoring the cytolytic effect of supernatants collected from U937Φ cells at 18 h after exposure to photosensitizer and light on the actinomycin D-sensitized L929 mouse fibroblast cell. L929 is a known TNF-α responsive cell line and is commonly used for the testing of biological activity of TNF-α. The data reported in the present section are for anti-TNF-α preventable cytolysis (see Materials and Methods).

The TNF-α-mediated toxicity on L929 cells exposed to the conditioned media from untreated macrophages was 20% (± 11%). Increased level of toxicity induced by the supernatant from untreated macrophages was expected since the U937 cells were differentiated into macrophages by TPA, which induces TNF-α production (Aderka et al, 1989). Toxicity in the presence of supernatants from macrophages treated with sensitizer alone did not differ significantly from that of controls.

As shown in Figure 3, conditioned media from photodynamically treated U937Φ cells induced cytolytic effect on L929 fibroblast cells. L929 cytolysis increased significantly after exposure to 2.3 mJ $cm^{-2}$. Maximum increase in cell cytolysis was observed after irradiation with 27.6 mJ $cm^{-2}$ and was 1.7-fold greater than in control (Figure 3). Afterwards cytolysis decreased progressively, reaching the baseline level after irradiation with the light fluence 0.2 J $cm^{-2}$. It should be noted that the level of PDT-generated TNF-α-independent component of L929 cell lysis was variable, but it did not differ significantly from that induced by culture medium from untreated macrophages.

### NO production by photodynamically treated U937Φ cells

In the present section we have studied NO generation from mTHPC-photosensitized macrophages. NO production was tested in macrophage supernatants 18 h after treatment by the conventional colourimetric assay.

Additional parallel set of control cultures consisted of untreated macrophages (neither photosensitizer nor light), tested under the same experimental conditions. The level of NO production by the untreated macrophages was 1.0 ± 0.2 μM and did not differ significantly from NO level released by macrophages exposed to photosensitizer alone. When cells were treated photodynamically, the level of NO in the macrophage supernatants increased gradually with an increase in light fluences (Figure 4), reaching a maximum (180%) after irradiation at 69 mJ $cm^{-2}$. Further increase in irradiation fluence caused the decrease in NO production, which still remained significantly higher than that in the untreated controls. Results in Figure 4 are expressed as the absorbance of photodynamically treated samples relative to the absorbance of the control culture exposed to photosensitizer alone.

### DISCUSSION

Photodynamic activation of macrophage-like cells contributes to an effective outcome of PDT treatment. Activated by photodynamic treatment macrophages become more efficiently involved in killing cancerous cells, as well as in phagocytosis of tumour cell debris (Dougherty, 1998). The ingestion of PDT-damaged or killed cells by macrophages may create conditions for the tumour antigen presentation by these cells, resulting in a possible development of tumour-specific immunity (Korbelik and Krosl, 1996). Consequently, PDT appears to be highly receptive to the adjuvant treatment with the macrophage-activating factors. For example, the effect of PDT treatment of tumours was shown to be enhanced by vitamin D3-binding protein-derived macrophage-activating factor (DBMAF) (Korbelik et al, 1997), glucan SPG (Krosl and Korbelik, 1994) as well as granulocyte–macrophage colony-stimulating factor (GM-CSF) (Krosl et al, 1996).

© 1999 Cancer Research Campaign

Photodynamic enhancement of macrophage phagocytosis has been reported for HpD (Yamamoto et al, 1991), Photofrin II (Yamamoto et al, 1994) and some cyanine dyes (Yamamoto et al, 1992*a*; Nakagawa et al, 1993). The results of the present study add to this list mTHPC, a potent second-generation photosensitizer. We have demonstrated that mTHPC and sublethal light doses enhance macrophage phagocytic capacity (Figure 1). In the above cited works, photodynamic activation of macrophage phagocytic capacity was studied in the model of Fc-receptor-mediated phagocytosis of the opsonized erythrocytes (Yamamoto et al, 1991, 1994). The enhanced macrophage activity was considered to be caused by lipid photoperoxidation reactions in co-present non-adherent lymphocytes with the subsequent release of macrophage-stimulating factors. In the present work, phagocytic activity of U937Φ cells was studied in the model of macrophages interaction with fluorescein conjugated *E. coli* particles. In this case phagocytosis proceeds via mannose-fucose receptors on the ingesting cells. Basically, these receptors recognize terminal mannose or fucose saccharides on the lipopolysaccharide of invading bacteria (Brown, 1995). Therefore, photosensitized by mTHPC enhancement of ingestion capacity may be mediated by activation of mannose-fucose receptors of U937Φ cells. The mechanism for such photodynamic induction of receptor activation is presently unclear. However, enhancement in phagocytic capacity can be induced by cytokines released from the photosensitized macrophages. For example, such cytokines as interferon (IFN)-γ, interleukin (IL)-4 and TNF-α are known to activate numerous macrophage functions (Collins and Bancroft, 1992). Indeed, the fact that under our experimental conditions mTHPC-photosensitized macrophages can produce TNF-α was demonstrated by our further experiments. As it is shown in Figure 3, light energy-dependent induction of TNF-α-mediated cytolysis by photodynamically treated macrophages was observed 18 h after irradiation. The initial increase in TNF-α-mediated cytolysis was taking place in the same range of light doses with phagocytosis activation. It is therefore possible that TNF-α production contributes to phagocytosis enhancement after mTHPC-PDT treatment of the macrophages. TNF-α-mediated cytolysis decreased after irradiation with the light fluences greater than 28 mJ $cm^{-2}$ and reached the untreated control level at 0.2 J $cm^{-2}$. Photocytotoxicity test employed under the same experimental conditions revealed increase in toxicity after much higher irradiation fluences (ca. 20% at 0.35 J $cm^{-2}$) (Figure 2). Such correlation is consistent with an earlier observation made by Evans et al (1990), who have demonstrated that macrophages become incapable of producing TNF before they are irreversibly damaged.

The relevance of these findings to clinical mTHPC-PDT remains to be clarified. However, we would expect that increase in phagocytosis could improve the PDT curative effect through the cancer cell elimination by activated macrophages. Biological significance of the TNF-α production after in vivo mTHPC-PDT may be of particular interest, since TNFs are not only associated with direct cytotoxicity on tumour cells, but also may mediate tumour regression through the effects on the microvasculature (Evans et al, 1990).

The important finding of our study is a fluence-dependent increase in NO release from the macrophages photosensitized with mTHPC and sublethal light doses (Figure 4). Compared to phagocytosis enhancement, where the highest fluence applied (0.2 J $cm^{-2}$) resulted in phagocytosis suppression below the base level, the NO release from photodynamically treated macrophages was still greater as compared to the untreated control. The increased level of NO production even at high irradiation fluences may probably explain phagocytosis suppression. Indeed, NO production was reported to exert significant negative effect on the phagocytic function on rat elicited macrophages (Albina and Reichner, 1998).

Finally, the relationship between phagocytosis activation, release of NO and cytokines production may be established by monitoring the macrophage phagocytic capacity in the presence of NO synthase inhibitors and TNF-α antibodies, and this serves as the source of ongoing investigations.

The implications of NO in the events participating in anti-tumour effects of PDT are now well-established (Korbelik et al, 1998). NO has been proposed to be the key molecule determining sensitivity to oxidative stress, including that induced by PDT, at the level of the vascular endothelium in tumours (Parkins et al, 1995; Korbelik et al, 1998). Nevertheless, only few studies report about PDT-induced NO production (Buettner and Kelley, 1997; Gupta et al, 1998). The NO generation by human carcinoma cells as a result of photosensitization with silicon-phtalocyanine was recently reported (Gupta et al, 1998). The authors proposed that NO might be involved in PDT-induced apoptosis (Gupta et al, 1998). Also, an increase in nitrosylhaemoglobin (• HO-NO) production in tumour tissue of mice after Photofrin-PDT was observed (Buettner and Kelley, 1997). The results of the present work demonstrated for the first time the generation of NO by photodynamically treated macrophages.

In our institution, mTHPC is being applied only for the treatment of early-stage (in situ and microinvasive) malignancies of the upper aerodigestive tract. Macrophages represent a large cell population in the early-stage tumours (Korbelik and Krosl, 1996). They are concentrated in lesion periphery and will, in all probability, be subjected to feeble irradiation fluences in course of PDT. Therefore, reported in the present work manifestations of activation of macrophage-like cells by mTHPC and subcurative light doses, are very likely to occur in clinical context.

### ACKNOWLEDGEMENTS

This work was supported by Alexis Vautrin Cancer Center Research Funds, French Ligue Nationale contre le Cancer (Lorraine Region Committee), and French Ministry of Foreign Affairs (scholarships to VM and IB).

### REFERENCES

Adams DO and Nathan CF (1983) Molecular mechanisms in tumor cell killing by activated macrophages. *Immunol Today* **4**: 166–170

Aderka D, Le JM and Vilcek J (1989) IL-6 inhibits lipopolysaccharide-induced tumor necrosis factor production in cultured human monocytes, U937 cells, and in mice. *J Immunol* **143**: 3517–3523

Albina JE and Reichner JS (1998) Role of nitric oxide in mediation of macrophage cytotoxicity and apoptosis. *Cancer Metastas Rev* **17**: 39–53

Anderson C, Hrabovsky S, McKinley Y, Tubesing K, Tang H, Dunbar R, Mukhtar H and Elmets CA (1997) Phtalocyanine photodynamic therapy: disparate effects of pharmacologic inhibitors on cutaneous photosensitivity and on tumor regression. *Photochem Photobiol* **65**: 895–901

Asher A, Mule JJ, Reichert CM, Shiloni E, Rosenberg SA (1987) Studies on the anti-tumor efficacy of systemically administered recombinant tumor necrosis factor against several murine tumors in vivo. *J Immunol* **138**: 963–974

Brown EJ (1995) Phagocytosis. *BioEssays* **17**: 109–117

Buettner GR and Kelley EE (1997) EPR detection of nitric oxide (NO) in the phototherapy of tumors. *Photochem Photobiol* **65**: 55S

© 1999 Cancer Research Campaign 

Collins HI and Bancroft GJ (1992) Cytokine enhancement of complement-dependent phagocytosis by macrophages: synergy of tumor necrosis factor-α and granulocyte-macrophage colony-stimulating factor for phagocytosis of *Cryptococcus neoformans. Eur J Immunol* **22**: 1447–1454

Dilkes MG, DeJode ML, Rowntree-Taylor A, McGilligan JA, Kenyon GS and McKelvie (1997) mTHPC photodynamic therapy for head and neck cancer. *Lasers Med Sci* **11**: 23–29

Dougherty TJ, Gomer CJ, Henderson BW, Jori G, Kessel D, Korbelik M, Moan J and Peng Q (1998) Photodynamic therapy. *J Natl Cancer Inst* **90**: 889–905

Evans S, Matthews W, Perry R, Fraker D, Norton J and Pass HI (1990) Effect of photodynamic therapy on tumor necrosis factor production by murine macrophages. *J Natl Cancer Inst* **82**: 34–39

Grosjean P, Savary J and Wagnieres G (1993) Tetra(m-hydroxyphenyl)chlorin clinical photodynamic therapy of early bronchial and oesophageal cancers. *Lasers Med Sci* **8**: 235–243

Gupta S, Ahmad N and Mukhtar H (1998) Involvement of nitric oxide during phthalocyanine (Pc4) photodynamic therapy-mediated apoptosis. *Cancer Res* **58**: 1785–1788

Henderson BW and Dougherty TJ (1992) How does photodynamic therapy work? *Photochem Photobiol* **55**: 145–157

Hoff T, Spencker T, Emmendoerffer A and Goppelt-Struebe M (1992) Effects of glucocorticoids on the TPA-induced monocytic differentiation. *J Leukoc Biol* **52**: 173–182

Korbelik M and Krosl G (1996) Photosensitizer distribution and photosensitized damage of tumor tissues. In: *The Fundamental Bases of Phototherapy*, Honigsmann H, Jori G and Young AR (eds), pp. 229–245: OEMF spa: Milan

Korbelik M, Naraparaju VR and Yamamoto N (1997) Macrophage-directed immunotherapy as adjuvant to photodynamic therapy of cancer. *Br J Cancer* **75**: 202–207

Korbelik M, Shibuya H, Cecic I (1998) Relevance of nitric oxide to the response of tumors to photodynamic therapy. In: *Optical Methods for Tumor Treatment and Detection: Mechanisms and Techniques in Photodynamic Therapy VII*, Dougherty TJ (ed), Proceedings of *SPIE* **3247**: 98–105

Krosl G and Korbelik M (1994) Potentiation of photodynamic therapy by immunotherapy: the effect of schizophyllan (SPG). *Cancer Lett* **84**: 43–44

Krosl G, Korbelik M and Dougherty GJ (1995) Induction of cell infiltration into murine SCCVII tumor by photofrin-based photodynamic therapy. *Br J Cancer* **71**: 549–555

Krosl G, Korbelik M, Krosl J and Dougherty GJ (1996) Potentiation of photodynamic therapy elicited antitumor response by localized treatment with granulocyte-macrophage colony stimulating factor. *Cancer Res* **56**: 3281–3286

McHale AP and McHale L (1988) Use of tetrazolium based colorimetric assay in assessing photoradiation therapy in vitro. *Cancer Lett* **41**: 315–321

Merlin JL, Azzi S, Lignon D, Ramacci C, Zeghari N and Guillemin F (1992) MTT assay allow quick and reliable measurement of the response of human tumor cells to photodynamic therapy. *Eur J Cancer* **29**: 1452–1458

Nakagawa Y, Homma S, Yamamoto I, Banno M, Nakazato H, Imanaga H and Yamamoto N (1993) In vivo and in vitro activation of macrophages with a cyanine photosensitizing dye, platonin. *Cancer Immunol Immunother* **37**: 157–162

Parkins CS, Dennis MF, Stratford MRL, Hill SA and Chaplin DJ (1995) Ischemia reperfusion injury in tumors: the role of oxygen radicals and nitric oxide. *Cancer Res* **55**: 6026–6029

Pass HI (1993) Photodynamic therapy in oncology. *J Natl Cancer Inst* **85**: 443–456

Ris HB, Altermatt HJ, Inderbitzi R, Heiss R, Nachbur B, Stewart JCM, Wang Q, Lim CK, Bonett R and Althaus U (1991) Photodynamic therapy with chlorins for diffuse malignant mesothelioma: initial clinical results. *Br J Cancer* **64**: 1116–1120

Sveinbjornsson B, Olsen R, Seternes OM and Seljelid R (1996) Macrophage cytotoxicity against murine Meth A sarcoma involves nitric oxide-mediated apoptosis. *Biochem Biophys Res Commun* **223**: 643–649

Wan CP, Choon SP and Lau BHS (1993) A rapid and simple microfluorimetric phagocytosis assay. *J Immunol Methods* **162**: 1–7

Yamamoto N, Homma S, Sery TW, Donoso LA and Hoober JK (1991) Photodynamic immunopotentiation: in vitro activation of macrophages by treatment of mouse peritoneal cells with hematoporphyrin derivative and light. *Eur J Cancer* **27**: 467–471

Yamamoto N, Homma S, Nakagawa Y, Hayami M, Imanaga H, Kurimoto M, Misuhashi M and Kimoto T (1992a) Activation of mouse macrophages by *in vivo* and *in vitro* treatment with a cyanine dye, lumin. *J Photochem Photobiol B: Biol* **13**: 295–306

Yamamoto N, Hoober K and Yamamoto S (1992*b*) Tumoricidal capacities of macrophages photodynamically activated with hematoporphyrin derivative. *Photochem Photobiol* **56**: 245–250

Yamamoto N, Sery TW, Hoober JK, Willett NP and Lindsay DD (1994) Effectiveness of photofrin II in activation of macrophages and in vitro killing of retinoblastoma cells. *Photochem Photobiol* **60**: 160–164

*© 1999 Cancer Research Campaign*
